# Prevalence of asymptomatic malaria, submicroscopic parasitaemia and anaemia in Korogwe District, north-eastern Tanzania

**DOI:** 10.1186/s12936-021-03952-3

**Published:** 2021-10-29

**Authors:** Paul M. Hayuma, Christian W. Wang, Edwin Liheluka, Vito Baraka, Rashid A. Madebe, Daniel T. R. Minja, Gerald Misinzo, Michael Alifrangis, John P. A. Lusingu

**Affiliations:** 1 National Institute for Medical Research, Tanga Research Centre, P. O. Box 5004, Tanga, Tanzania; 2 Department of Veterinary Microbiology, Parasitology and Biotechnology, College of Veterinary Medicine and Biomedical Sciences, Sokoine University of Agriculture, P. O. Box 3019, Morogoro, Tanzania; 3 Department of Immunology and Microbiology, Centre for Medical Parasitology, University of Copenhagen, Copenhagen, Denmark; 4 Department of Infectious Diseases, Copenhagen University Hospital, Copenhagen, Denmark; 5 SACIDS Africa Centre of Excellence for Infectious Diseases, SACIDS Foundation for One Health, Sokoine University of Agriculture, P. O. Box 3297, Morogoro, Tanzania

**Keywords:** *Plasmodium falciparum*, Asymptomatic infection, Anaemia, Submicroscopic parasitaemia, North-eastern Tanzania

## Abstract

**Background:**

Asymptomatic malaria infections largely remain undetected and act as a reservoir for continuous transmission. The study assessed the prevalence of submicroscopic asymptomatic malaria infections and anaemia in two rural low (300 m above sea level) and highland (700 m asl) settings of Korogwe District north-eastern Tanzania.

**Methods:**

A cross-sectional malariometric survey involving individuals aged 0–19 years was conducted in June 2018 in the two rural villages. Venous blood was collected from eligible study participants for estimation of haemoglobin level, detection of malaria by rapid diagnostic test (RDT), quantification of malaria parasitaemia by microscopy, as well as dried blood spot (DBS) for determining submicroscopic infections by PCR targeting the small subunit of the ribosomal ribonucleic acid (ssrRNA) of human *Plasmodium.*

**Results:**

Out of 565 individuals tested, 211 (37.3%) were malaria positive based on RDT, whereas only 81 (14.3%) were positive by microscopy. There was no significant difference in the prevalence between the highland and the lowland village, p = 0.19 and p = 0.78 microscopy and RDT, respectively. Three out of 206 (1.5%) RDT/microscopy negative samples were *P. falciparum* positive by PCR. Of the 211 RDT and 81 microscopy positive, 130 (61.6%) and 33 (40.7%), respectively, were defined as being asymptomatic. Of the 565 individuals, 135 (23.9%) were anaemic (haemoglobin < 11 g/dL) out of which 5.2% were severely anaemic. The risk of being anaemic was significantly higher among individuals with asymptomatic malaria as compared to those without malaria as confirmed by RDT (AOR = 2.06 (95% CI 1.32–3.20) while based on microscopic results there was no significant differences observed (AOR = 2.09, 95% CI 0.98–4.47). Age and altitude had no effect on the risk of anaemia even after adjusting for asymptomatic malaria.

**Conclusions:**

Asymptomatic malaria is associated with an increased risk of having anaemia in the study communities. The findings highlight the need for targeted interventions focusing on asymptomatic infections which is an important risks factor for anaemia in the community and act as a source of continued transmission of malaria in the study area.

## Background

Malaria claims lives of more than 400,000 people annually [1]. Reportedly, there has been a global reduction in malaria cases from 238 to 229 million between 2000 and 2019, but the sub-Saharan Africa (SSA) still experiences an unprecedented disease burden attributable to malaria [1]. The existing malaria control tools including the deployment of effective anti-malarial drugs, such as artemisinin-based combination therapy (ACT) has substantially contributed in curtailing transmission through its gametocytocidal effect and hence influenced the observed reduction in malaria related morbidities and mortalities [2].

In Tanzania, in addition to ACT use, successful implementation of other malaria preventive strategies including wide use of long-lasting insecticidal nets could most likely explain the observed decline in malaria prevalence in the general population from 18 to 7% between 2008 and 2017 [3]. Various epidemiological studies conducted in north-eastern Tanzania have reported a significant decline in malaria between 2003 and 2012 [4–7] followed by a temporal increase in 2016 and 2017 [4, 5].

In malarial endemic settings, repeated infections cause a partial non-sterilizing immunity to malaria resulting into asymptomatic and often, chronic infections that are rarely detected and may end up not being treated [8]. Many of these asymptomatic malaria carriers maintain low level parasitaemia and gametocytaemia which may play a significant role in transmission [9]. Moreover, even low level malaria parasitaemia is associated with an increased risk of anaemia [10, 11] and may impair cognitive development [12].

Detection of malaria parasites by microscopy (gold standard) or rapid diagnostic test (RDT) are challenged by a number of factors including limited availability of expert microscopists, low detection rates, especially when the parasitaemia is too low and high level false positive rates with RDT due to circulating parasite antigens [13]. The use of molecular techniques that detect parasite DNA offers an important research tool that can reveal submicroscopic parasitaemia undetectable by these conventional malaria diagnostic tools [8].

Despite the realized success in malaria control from various control interventions that have resulted into a significant decline in malaria related morbidity and mortality in north-eastern Tanzania [6–8], there is still paucity of data on the prevalence of asymptomatic malaria infections and its association with anaemia in this malaria endemic setting. The present study aimed to determine the prevalence of asymptomatic, submicroscopic malaria infections and anaemia in two rural communities of Kwamasimba (highland village) and Mkokola (lowland village,) in Korogwe District in north-eastern Tanzania.

## Methods

### Study sites

This cross-sectional study was conducted in two villages of Korogwe District, namely Mkokola (a village in a lowland area, 300 m asl) and Kwamasimba (a highland village, 700 m asl) in north-eastern Tanzania. The prevalence of *P. falciparum* malaria parasitaemia in the lowland village has been on decline from 78% to 2003 to 13% in 2008, whereas in the highland village, the prevalence decreased from 25 to 3% in the same period [7]. Subsistence farming and informal petty trading are the main income generating activities in this rural setting. The data presented here is of over 10 years as this data come from a continuous malaria project in the same study area from 2003 to date. The data published in public domain of is of 2003 to 2008. The available unpublished data of 2009 to 2018 has different trends of malaria prevalence in the study area, this data will be published later.

### Study design and population

This study collected data from a cross-sectional malariometric survey as part of the main study entitled “Malaria Research Capacity building for field clinical trial in Tanzania” (MaReCa Project-TMA 2015 SF-998) in two study villages. Analysed data included malaria epidemiological data collected in specially designed questionnaires (clinical, demographic and socioeconomic data) from individuals aged 0–19 years. Laboratory data were obtained from RDT, haemoglobin measurements by HemoCue machines, and typing for submicroscopic parasitaemia by PCR from parasite DNA extracted from dried blood spots (DBS). The prevalence of submicroscopic infection was determined from DBS that were selected based microscopic negative blood smears and were as well RDT negative. The DBS (n = 206, 43%) were selected from RDT and blood smears negatives to determine the prevalence of submicroscopic infections.

### Malariometric survey

The cross-sectional malariometric survey was conducted in June 2018 soon after cessation of the long rainy season with concomitant peak malaria transmission. Socio-demographic information including age, gender and place of residence were recorded using standardized questionnaires. Axillary body temperature was measured using digital thermometers (Omron, Turkey). Symptoms related to malaria such as fever, vomiting, convulsion, headache, loss of appetite and general body malaise were also recorded. Also study participants were asked if they have been taken any anti-malarial treatment within 14 days prior to enrollment into the study. Venous blood was collected in ethylenediaminetetraacetic acid (EDTA) vacutainers and blood remaining in the butterfly tube was used to prepare DBS on Whatman filter paper (Whatman #3, WVR, Søborg, Denmark), dried at room temperature and stored in silica gel). Malaria was tested by using thick and thin blood smears microscopy, as well as RDT (Care Start^TM^, Somerset, NJ USA) with both histidine rich protein 2 (HRP-2) and parasite lactate dehydrogenase (pLDH) following the manufacturer’s instructions. Haemoglobin (Hb) measurement was done in the field using HemoCue 301 machine (Angelholm, Sweden). All participants with uncomplicated malaria based on RDT and other clinical algorithms as per clinician discretions were treated using artemether–lumefantrine (AL) according to the existing Tanzanian malaria treatment guidelines [14]. However, for severely sick patients referrals to the nearby health facility were made as appropriate.

### Microscopic examination of thick and thin blood smears

Thin blood smears for the detection and quantification of malaria parasites were fixed with absolute methanol. Both thick and thin smears were then stained with 10% Giemsa for 30 min, gently washed with tap water, air dried and examined at 100× objectives using a binocular microscope (CX23 Olympus Corporation, Tokyo, Japan). Asexual parasites were counted against 200 leukocytes and sexual parasite counts were done against 500 leukocytes. If the asexual parasite counts were less than 10, reading was done per 500 leukocytes. All slides were read twice by two independent, certified microscopists and results from the two readings with a difference of less than 50% were considered definitive. Smears with discordant results were re-examined by a third reader blinded to the first and second readings. Results from the two independent readers that were in agreement were considered final. A slide was declared negative if no parasites were seen after scanning 200 high power fields. All microscopists who read the blood slides participated in proficient microscopic examination and were certified by the National Institute for Communicable Diseases (NICD), South Africa.

### Genomic DNA extraction and molecular detection of malaria parasites by PCR

Malaria parasite DNA was extracted from randomly selected DBS that were collected from RDT negative individuals and confirmed to be negative by microscopy. The DNA was extracted using QIAamp DNA Blood mini kit (Qiagen GmbH, Hilden, Germany), as per manufacturer’s instructions. After extraction, the eluted DNA was dispensed into 1.5 mL Eppendorf tubes and stored at − 20 °C. One microlitre of parasite genomic DNA was amplified using a thermocycler (T Professional, Thermocycler Biometra GmbH, Saxony, Germany). Detection submicroscopic infections of *Plasmodium* species was done by *Plasmodium* genus and species-specific PCR targeting the small subunit of the ribosomal ribonucleic acid (ssrRNA) genes of human *Plasmodium* using conditions as previously described [15]. Briefly, the master-mix reagents per sample comprised of; 1 µL of primer mix (forward and reverse primers 0.25 nM), 10 µL of Tempase and 8 µL of PCR quality water. Targeted species were *Plasmodium falciparum, Plasmodium malariae* and *Plasmodium ovale. Plasmodium falciparum* forward and reverse primers 5′-TTA AAC TGG TTT GGG AAA ACC AAA TAT ATT-3′, 5′-ACA CAA TGA ACT CAA TCA TGA CTA CCC GTC-3′, respectively. *Plasmodium malariae* forward and reverse primers 5′ AAA ATT CCA TGC ATA AAA TTA TAC AAA-3′, 5′-ATC TCT TTT GCT ATT TTT TAG TAT TGG AGA-3′, respectively. *Plasmodium ovale* forward and reverse primers 5′-ATC TCT TTT GCT ATT TTT TAG TAT TGG AGA-3′, GGA AAA GGA CAT TAA TTG TAT CCT AGTG-3′, respectively.

For each species targeted positive and negative controls were run together with the extracted DNA samples. To ensure quality of the PCR, positive and negative controls were included in each set of PCR run. As positive control for *P. falciparum* 3D7 strain and DNA extracted from DBS collected from microscopy confirmed *P. falciparum* positive cases whereas for *P. malariae* and *P. ovale* parasite DNA was extracted from microscopy confirmed *P. malariae* and *P. ovale cases.* Negative controls used was ultrapure water. Ethidium bromide stained 2% agarose gel was used to resolve amplicons under Ultra Violet-BioDoc Analyzer (Biometra GmbH, Saxony, Germany).

### Data management and analysis

Data were entered and validated using a double entry system in Microsoft access (Version 2007). Data analysis was done using STATA statistical software package version 13.1 (Stata Corp College Station, Texas USA). Proportions between groups were compared using χ^2^-test. Univariate and multivariate logistic regression analyses were used to assess the associations between anaemia with asymptomatic malaria, gender, altitude and age. Effects sizes were presented using odds ratio (OR) with 95% confidence intervals (CI). P-value of < 0.05 was considered statistically significant.

### Case definitions

The study enrolled individuals aged 0–19 years with permanent residence in the study area. These individuals (< 19 years) willingly consented to participate or their parents/guardians gave an informed consent for their participation. Individuals with positive RDT/microscopy and temperature above 37.5 °C and as well with signs of malaria, such as vomiting, convulsion, general body malaise, headache and/or loss of appetite, were considered symptomatic. Asymptomatic malaria individuals were defined as those individuals who had axillary body temperature below 37.5 °C, no history of fever for the past 48 h and were malaria positive by RDT, microscopy or PCR [16]. Submicroscopic malaria carriers were defined as individuals who were malaria positive based on PCR but were both microscopy and RDT negative. Anaemia was defined according to the World Health Organization (WHO) age-adjusted cut-off for haemoglobin (Hb) levels [17], whereby a Hb level < 7.0 g/dL was regarded as severe anaemia, between 7.0 and 9.9 g/dL as moderate anaemia, between 10.0 and 10.9 g/dL as mild anaemia and Hb > 11 g/dL as non-anaemic.

## Results

### Baseline characteristics of the study participants

A total of 565 study participants were enrolled of whom 280 were from the highland village and the remaining (n = 285) were from the lowland village. The majority of the participants were children aged below 5 years (42.0%), followed by 5–9 years (25.7%) and the least were those aged 17 –19 years (11.5%), Table 1.

**Table 1:** Demographic information of study participants from Kwamasimba (highland) and Mkokola (lowland) villages, Korogwe District, north-eastern Tanzania

Variable	Number of participants	Percentage (%)
Strata		
Highland village, Kwamasimba	280	49.6
Lowland village, Mkokola	285	50.4
Sex		
Male	275	48.8
Female	290	51.2
Age groups (years)		
0–4	237	42.0
5–9	145	25.6
10–14	118	20.9
15–19	65	11.5

### Prevalence of asymptomatic malaria and submicroscopic parasitaemia in highland and lowland villages

**Fig. 1:**
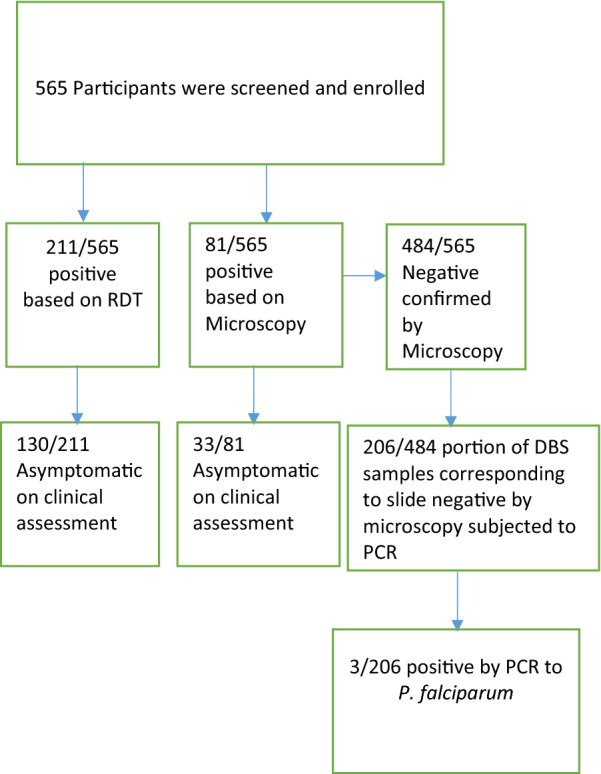
Consort diagram showing sampling strategies, data collection and downstream analyses

The overall malaria point prevalence in the two villages was 37.3% (211/565) as determined by RDT, but with almost 2.5-fold drop in prevalence 14.3% (81/565) as revealed by microscopy (Table 2). Upon stratification by altitude, the highland village showed an overall malaria point prevalence of 38.2% (107/280) and 18.6% (52/280) based on RDT and microscopy, respectively. On the other hand, in the lowland village the overall malaria point prevalence stood at 36.5% (104/285) based on RDT and 10.2% (29/285) by microscopy (Table 2). Among the 211 positive based on RDT results 38.4% (81/211) were symptomatic whereas based on microscopy results out 81 positive 59.3% (48/81) where symptomatic. Of the 211 RDT and 81 microscopy positive, 61.6% (95% CI 54.9–68.2%, (130/211) and 41.0% (95% CI 28.6–50.4%, (33/81) were defined as asymptomatic malaria infections, respectively. Again upon stratification by location, 63/280 (22.5%) and 20/280 (7.1%) in the highland villages were asymptomatic based on RDT and microscopy, respectively. On the other hand, in the lowland village, the prevalence was 67/285 (23.5%) and 13/285 (4.6%) based on RDT and microscopy, respectively (Table 3). It was observed that 133/211 (63.0%) of the RDT positive were microscope negative. The proportion of malaria was significantly higher among those aged above 5 years of age (by microscopy and RDT) than those aged below five (*p *< 0.01) (Table 2). *P. falciparum* was the most predominant malaria parasite detected by RDT and microscopy, 92.5% and 98.8%, respectively. *Plasmodium malariae* was detected only in one sample by microscopy. Of the microscopically positive samples, none was infected with a mixture of *Plasmodium* species. Only 0.85% (3/354) of the RDT negatives samples were found slide positive. *Plasmodium falciparum* gametocytes were detected in 6.2% (5/81) of the samples by microscopy. The prevalence of submicroscopic infection based on PCR was 1.5% (3/206) (Fig. 1). None of the other *Plasmodium* species (*P. malariae* and *P. ovale*) were detected by PCR. The results indicated that the majority of the study participants 10.6% (60/565) who mentioned the use of anti-malarial within the past 2 weeks have taken ALU, while one participant have taken sulfadoxine–pyrimethamine.

**Table 2:** Overall prevalence of malaria in the highland and lowland based on malaria rapid diagnostic tests results and microscopy results

Variable	RDT		p-value	Microscopy		p-value
	Positive (%)	Negative (%)		Positive (%)	Negative (%)	
Age groups						
0–4 years ( n= 237)	62 (26.27)	175 (73.73)	**< 0.01**	15 (6.38)	222 (93.62)	**< 0.01**
5–9 years (n= 145)	53 (36.55)	92 (63.45)		20 (13.79)	125 (86.21)	
10–14 years (n= 118)	67 (43.2)	51 (56.8)		33 (27.97)	85 (72.03)	
15–19 years (n= 65)	29 (44.6)	36 (55.4)		13 (20.00)	52 (80.00)	
Strata						
Highland village (n = 280)	107 (38.21)	173 (61.79)	0.696	52 (18.57)	228 (81.43)	**0.05**
Lowland village (n= 285)	104 (36.62)	181 (63.38)		29 (10.25)	256 (89.75)	
Total (N= 565)	211 (37.41)	354 (62.59)		81 (14.39)	484 (85.61)	

*p* < 0.01 the proportion of malaria significantly high in children above five years by both RDT and microscopy and *p* = 0.05 the prevalence of malaria in the borderline between the highland and lowland village (in bold)

**Table 3:** Asymptomatic malaria infection by age groups and strata as detected by malaria rapid diagnostic test and microscopy

Variable	n	RDT		p-value	Microscopy		p-value
		Positive (%)	Negative (%)		Positive (%)	Negative (%)	
Age groups (years)							
0–4	237	40 (16.9)	197 (83.1)		6 (2.53)	231 (97.5)	
5–9	145	34 (23.6)	111 (76.6)	**< 0.01**	10 (6.9)	135 (93.1)	**< 0.01**
10–14	118	38 (32.2)	80 (67.8)		13 (11.0)	105 (89.0)	
15–19	65	18 (27.7)	47 (72.3)		4 (6.2)	61 (94.0)	
Strata							
Highland village	280	63 (22.5)	217 (77.5)		20 (7.1)	260 (93.0)	
Lowland village	285	67 (23.5)	218 (76.5)	0.78	13 (4.6)	272 (95.4)	0.19
Total	565	130 (23.0)	435 (77.0)		33 (5.8)	532 (94.2)	

*p* < 0.01 the proportion of asymptomatic malaria significantly high in the age group of 5–9 years by both RDT and microscopy compared to other age groups (in bold)

### Prevalence of anaemia

Of the 565 individuals screened, 135 (23.9%) were anaemic (Hb < 11 g/dL). Among the anaemic cases, 78/135 (57.8%) had mild anaemia, 50/135 (37.0%) had moderate anaemia and 7/135 (5.2%) were severely anaemic. There was no significant difference in the prevalence of anaemia between the low- and highland villages. There was no statistically significant difference in anaemia statuses between males and females (*p *= 0.25). Of the anaemic cases, anaemia seemed to vary with age groups but the difference was not statistically significant (*p *= 0.22) (Table 4).

**Table 4:** Anaemia status of the study participants by gender, age groups and strata

Variable	Non anaemic	Anaemia			Total n	P-value
	n (%)	Mild, n (%)	Moderate n (%)	Severe n (%)		
Sex						
Male	199 (72.4)	44 (16.0)	28 (10.1)	4 (1.50)	275 (48.7)	0.25
Female	231 (79.7)	34 (11.7)	22 (7.6)	3 (1.03)	290 (51.3)	
Age groups (years)						
0.5–4	174 (73.4)	43 (18.1)	17 (7.2)	3 (1.3)	237 (42.0)	0.22
5–11	153 (76.9)	19 (9.6)	23 (11.5)	4 (2.0)	199 (35.2)	
12–14	52 (81.3)	7 (10.9)	5 (7.8)	0 (0.0)	64 (11.3)	
15–19	51 (78.5)	9 (13.9)	5 (7.6)	0 (0.0)	61 (11.5)	
Strata						
Highland village	217 (77.5)	38 (13.6)	22 (7.9)	3 (1.0)	280 (49.7)	0.82
Lowland village	213 (74.7)	40 (14.0)	28 (9.8)	4 (1.4)	285 (50.3)	
Total	430 (76.1)	78 (13.8)	50 (8.9)	7 (1.2)	565 (100)	

Haemoglobin level < 7.0 g/dL = severe anaemia, between 7.0 and 9.9 g/dL = moderate anaemia, between 10.0 and 10.9 g/dL = mild anaemia and Hb ≥ 11 g/dL = non-anaemic. The age groups presented here are as per WHO haemoglobin concentrations for the diagnosis of anaemia and assessment of severity [17]

### Association between asymptomatic malaria and anaemia

The associations between asymptomatic malaria and anaemia (Hb < 11 g/dL), gender, age and altitude are explored in Table 5. Model 1 included asymptomatic malaria based on microscopy adjusted for gender, age and altitude. Model 2 included asymptomatic malaria based on RDT adjusted for gender, age and altitude. The risk of anaemia was significantly higher among the participants with asymptomatic malaria based on RDT (AOR= 2.06; 95% CI 1.32–3.20) adjusted for gender, age and altitude. Also based on microscopy results, participants with asymptomatic malaria had risk of anaemia, though not statistically significant (AOR= 2.09; 95% CI 0.98–4.47). Males with asymptomatic malaria seemingly had higher risk of having anaemia by 34% when based on RDT (AOR = 1, 95% CI 0.45–0.99) but was not significantly based on microscopy (AOR= 0.68; 95% CI 0.46–1.01). There were no statistically significant differences between having asymptomatic malaria on explanatory variables of altitude and age groups and the risk of having anaemia.

**Table 5:** Univariate and multivariate associations of anaemia with asymptomatic malaria using microscopy, RDT, adjusted for age, sex and altitude

Variable	Unadjusted OR 95% CI	p-values	Model 1	P-values	Model 2	P-values
			Adjusted OR, 95% CI		Adjusted OR, 95% CI	
Asymptomatic based on microscopy						
Negative	Ref		Ref	–	–	–
Positive	1.90 (0.90–4.0)	0.09	2.1 (1.0–4.5)	0.56	–	–
Age group (yr)						
< 5	Ref		Ref		Ref	
5–11	0.8 (0.5–1.3)	0.41	0.8 (0.5–1.2)	0.25	0.8 (0.5–1.2)	0.21
12–14	0.6 (0.3–1.3)	0.20	0.6 (0.3–1.2)	0.13	0.6 (0.3–1.1)	0.11
15–19	0.8 (0.4–1.5)	0.41	0.7 (0.4–1.5)	0.38	0.7 (0.4–1.4)	0.30
Sex						
Male	Ref		Ref		Ref	
Female	0.7 (0.5–1.0)*		0.7 (0.5–1.0)	0.53	1.0 (0.1–1.0)*	0.04
Altitude						
High	Ref		Ref		Ref	
Low	1.2 (0.8–1.7)	00.44	1.2 (0.8–1.8)	0.40	1.2 (0.8–1.7)	0.49
Asymptomatic based on RDT						
Negative	Ref	–	–	–	Ref	
Positive	1.9 (1.3–2.8)*	0.03	–	–	2.1 (1.3–3.2)*	0.01

*p < 0.05, Model 1 included asymptomatic malaria based on microscopic results adjusted for gender, age and altitude whilst model 2 included asymptomatic malaria based on RDT adjusted for gender, age and altitude. The age groups presented here are as per the WHO haemoglobin concentrations for the diagnosis of anaemia and assessment of severity [17]

## Discussion

The present study revealed a high prevalence of asymptomatic malaria in this rural population of north-eastern Tanzania. Interestingly, children aged above 5 years who are usually forgotten in many malaria control interventions were particularly vulnerable. It is high time now that instead of focusing on underfive and pregnant women, targeted malaria control interventions also consider school aged children who seem to be most vulnerable. Such a high proportion of asymptomatic malaria among those aged above 5 years suggests that school aged children (and above) are a major reservoir of malaria which is in line with other malaria studies from school aged children in Tanzania [18–20]. This raises an alarm for having targeted malaria control programmes for this particular group as these asymptomatic infections may result into persistently low level parasitaemia that may precipitate into anaemia and hence affect cognitive ability among school aged children.

As previously reported [22], the majority of infections were due to *P. falciparum* which is implicated for the more severe form of malaria pathogenesis. However, there were very few infections with *P. malariae*, and a *P. ovale* infection was not detected.

As expected, the study has noted remarkable differences in the detection of malaria by using microscopy and RDT. Notwithstanding, a better picture would have been possible if all RDT positive and all microscopy negative results could be subjected to species diagnostic PCR in order to rule out chances for false positives due to circulating parasite antigens or inability of detection by microscopy for submicroscopic infections. However, upon subjecting a calculated portion of both RDT and microscopy negative samples to malarial species diagnostic PCR, only 1.5% of the negative samples were confirmed to be sub-microscopic infections that were missed by both RDT and microscopy. Unfortunately, due to limitations of the study we did not have the opportunity to explore all RDT positive/microscopy negative samples by PCR. By PCR the current study analysed a subset of samples that were both negative by RDT and microscopy, this might have an impact on the results for submicroscopic as there were possibility of DBS selected to have false negative results by RDT as results of *P. falciparum* HRP2 gene deletion. The observed percentage of RDT negative which were found slide positive was very low hence chances of having HRP2 gene deleted samples in PCR analyses for submicroscopic was very low as well. Moreover, the current information reported on *P. falciparum* HRP2 gene deletion in Tanzanian community surveys shows HRP2 gene deletion is uncommon [32].

From this study, it is most likely that RDT may have overestimated the actual malaria point prevalence in the study area as previously reported elsewhere [13, 21, 23, 24]. Apart from being asymptomatic cases, without having PCR or equally sensitive or superior test, it would not be possible to have correct evaluation of the actual point prevalence based on RDT only as some of the positive cases could be false positives and this is one of the weaknesses for the current study.

It is worth mentioning, at least from an epidemiological viewpoint, despite being false positive and having the loophole for overestimating the actual point prevalence, in a resource limited setting like in Korogwe Tanzania, the use of RDT still provides some important information about current/recent infection and could guide attending clinician on what management to offer while ruling out the possibility of having malaria infection at least for those who are RDT negative. The challenge with the HRP-2-based RDTs is the detection of circulating *P. falciparum* HRP-2 in the blood stream for at least 2 weeks even after successful clearance of infected erythrocytes [25].

The current study observed no significant differences in the prevalence of asymptomatic malaria between the high- and lowland villages as revealed by either RDT and/or microscopy results. This finding is in contrary with a previous observation from the same study area in the period of 2003–2008 [7], however, this may be due to a combination of factors such as climate and environmental changes [26–28].

The overall prevalence of anaemia in this study, defined as Hb level < 11 g/dL was found to be in the category of moderate anaemia (20.0–39.9%). According to the WHO criteria on assessment for severity of anaemia [17], this finding implies that the study participants in this study area were significantly anaemic and raises some concerns. Individuals from the lowland village were at higher risk of having anaemia as compared to those from the highland, this phenomenon is in agreement with what has been reported previously from the same study sites [7]. Anaemia was associated with the asymptomatic malaria cases as diagnosed by RDT and microscopy which is in line with a previous study from Tanzania [29]. Other factors such as hookworm infestation schistosomiasis and nutritional factors were not assessed and these could have an influence on the observed prevalence of anaemia. With the absence of targeted malaria control efforts among school age children that is usually left out in many malaria control interventions, it is worrisome if this particular group is not included, all malaria control efforts might be highly compromised.

In this study, only few *P. falciparum* gametocytes were observed by microscopy which is contrary to previous studies that have shown a high prevalence of gametocyte carriage in settings with high burden of asymptomatic infections [30]. Therefore, sensitive diagnostic tools for the detection of submicroscopic gametocytes in this setting may be required in order to have a proper guidance for future implementation of strategies aimed at interrupting and curtailing transmission by targeting high-risk transmission hotspots through community-wide mass drug administration (MDA), seasonal malaria chemoprevention (SMC), mass screening and treatment campaigns (MSTC) and/or the use of single low dose of primaquine in elimination programmes [31].

## Study limitations

The submicroscopic component of the study analysed DBS samples by PCR that were all RDT negative. This might have an impact on results presented for submicroscopic as there where possibility of false negatives due to *pfhrp2* gene deletion.

## Conclusions

In this study, asymptomatic malaria has been shown as a risk factor for having anaemia in the two study communities of north-eastern Tanzania. The findings highlight the need for targeted interventions focusing on asymptomatic infections which sustain the potential for continued transmission of malaria in the study area. Furthermore, the fact that children above 5 years of age were particularly vulnerable to malaria as opposed to under-fives warrants for the introduction and rolling out targeted malaria control interventions focusing on school aged children.

## Data Availability

The dataset generated and/or analysed during the current study are available from the corresponding author on reasonable request.

## Declarations

The finding and conclusion presented in this report are those of the authors based on the data presented which was part of the main ongoing MaReCa Project in north-eastern Tanzania.
